# Omega-3 fatty acids impair miR-1-3p-dependent Notch3 down-regulation and alleviate sepsis-induced intestinal injury

**DOI:** 10.1186/s10020-021-00425-w

**Published:** 2022-01-28

**Authors:** You-Lian Chen, Yin-Jing Xie, Zhen-Mi Liu, Wei-Bu Chen, Ru Zhang, Hong-Xing Ye, Wei Wang, Xue-Yan Liu, Huai-Sheng Chen

**Affiliations:** 1 Department of Critical Care Medicine, Shenzhen People’s Hospital, The Second Clinical Medical College of Jinan University, The First Affiliated Hospital of South University of Science and Technology, No. 1017, Dongmen North Road, Luohu District, Shenzhen, 518020 Guangdong Province People’s Republic of China; 2 Clinical Laboratory, Shenzhen People’s Hospital, The Second Clinical Medical College of Jinan University, The First Affiliated Hospital of South University of Science and Technology, Shenzhen, 518020 People’s Republic of China; 3 Department of Gastroenterology, Shenzhen People’s Hospital, The Second Clinical Medical College of Jinan University, The First Affiliated Hospital of South University of Science and Technology, Shenzhen, 518020 People’s Republic of China; 4 Department of Endocrinology and Metabolism, Shenzhen People’s Hospital, The Second Clinical Medical College of Jinan University, The First Affiliated Hospital of South University of Science and Technology, Shenzhen, 518020 People’s Republic of China

**Keywords:** Omega-3 fatty acids, microRNA-1-3p, Notch3, Smad, Sepsis, Intestinal injury, Inflammation, Stress oxidative

## Abstract

**Background:**

Sepsis is a troublesome syndrome that can cause intestinal injury and even high mortality rates. Omega-3 fatty acids (FAs) are known to protect against intestinal damage. Accordingly, the current study set out to explore if omega-3 FAs could affect sepsis-induced intestinal injury with the involvement of the microRNA (miR)-1-3p/Notch3-Smad axis.

**Methods:**

First, cecal ligation and perforation (CLP) was performed to establish septic mouse models in C57BL/6J mice, and mouse intestinal epithelial MODE-K cells were induced by lipopolysaccharide (LPS) to establish sepsis cell models. The CLP-induced septic mice or LPS-exposed cells were subjected to treatment with Omega-3 FAs and activin (Smad signaling activator), miR-1-3p inhibitor and over-expressed/short hairpin RNA (oe-/sh)-Notch3 to explore their roles in inflammation, intestinal oxidative stress and cell apoptosis. A dual-luciferase reporter gene assay was further performed to verify the regulatory relationship between miR-1-3p and Notch3.

**Results:**

Omega-3 FAs inhibited CLP-induced intestinal injury and ameliorated LPS-induced intestinal epithelial cell injury by down-regulating miR-1-3p, as evidenced by decreased levels of tumor necrosis factor-α, interleukin-1β (IL-1β) and IL-6, in addition to diminished levels of reactive oxygen species, malondialdehyde levels and superoxide dismutase activity. Furthermore, miR-1-3p could down-regulate Notch3, which inactivated the Smad pathway.

**Conclusion:**

Collectively, our findings indicated that omega-3 FAs elevate the expression of Notch3 by down-regulating miR-1-3p, and then blocking the Smad pathway to alleviate intestinal epithelial inflammation and oxidative stress injury caused by sepsis.

**Supplementary Information:**

The online version contains supplementary material available at 10.1186/s10020-021-00425-w.

## Background

Sepsis is a highly-complicated disease of infection-provoked serious systemic inflammation, that can result in organ dysfunction and considerably high mortality (Venet and Monneret 2018; Lelubre and Vincent 2018). Adding to the plight of the patients, systemic inflammatory response syndrome and multiple organ dysfunction syndrome can further lead to acute gastrointestinal injury (Chen et al. 2015). Despite the tremendous strides made in the treatment of sepsis in the form of fluid/metabolic resuscitation, hemodynamic and end-organ aid, as well as the use of antibiotics, the high mortality still exerts a heavy burden on medical infrastructure all over the globe (Perner et al. 2017; Yuk et al. 2018). Nevertheless, the hard work of our fellow researchers has identified the intestine as one of the major targets of sepsis, and thus identification of protective strategies for sepsis-induced injury to intestinal mucosa crossed our minds (Chang et al. 2013). Meanwhile, oxidative stress triggered by commonly-occurring inflammatory responses in sepsis is also known to precipitate organ damage (Galley 2011). In this context, it would be pragmatic to seek out novel pathways to control the sepsis-induced intestinal injury which is primarily associated with inflammation and oxidative stress.

It is no surprise that omega-3 fatty acids (FAs) are regarded as important dietary nutrients, due to their critical value in the structure and functioning of the nervous system (Darcey and Serafine 2020). Intriguingly, administration of omega-3 FAs was previously highlighted as a safe and efficacious therapy to alleviate organ failure evoked by sepsis (Wolbrink et al. 2020). Additionally, another study suggested that omega-3 lipid emulsion could diminish oxidative stress in rats with intestinal ischemia–reperfusion injury (Arisue et al. 2012). Furthermore, omega-3 polyunsaturated fatty acids are known to confer protective effects against intestinal damage in rats suffering from peritoneal dialysis (Tang et al. 2019).

On a separate note, microRNAs (miRs), which are widely-regarded as important regulators of gene expression through repression of translation or degradation of messenger RNA (mRNA), have unsurprisingly been implicated in the development of sepsis (Essandoh and Fan 2014). For instance, miR-1-5p, one such miR, exerts a critical role in aggravating lipopolysaccharide (LPS)-induced sepsis in mouse models (Han et al. 2020). In addition, miR-1 can target the Notch3 gene to promote myocardial ischemia injury (Xu et al. 2020). Similarly, miR-1 is capable of down-regulating Notch3 in differentiating myoblasts to deteriorate hypoxic injury (Gagan et al. 2012). Meanwhile, recent studies have also suggested that the Notch signaling pathway possesses the ability to ameliorate lung injury in septic rats (Zhang et al. 2020). Furthermore, Notch3 activation can confer satisfactory protection against hypertension-associated heart failure via mediation of oxidative stress (Ragot et al. 2016). It is also noteworthy that Notch3 can inhibit the activation of Smad3 signal to reduce cardiac fibrosis (Zhang et al. 2016). This is particularly important as the Smad protein family is known to transduce signals from transforming growth factor (TGF) ligands, which are capable of mediating cell behaviors via receptor serine/threonine kinase activation (Singh et al. 2011). More importantly, suppression of Smad2/3 phosphorylation has been previously shown to ameliorate radiation-induced intestinal injury in mouse models (Kim et al. 2017). Initial findings in our study further suggested there could be regulatory relationship between miR-1-3p and omega-3 FAs. In lieu of the aforementioned findings, we hypothesized that omega-3 FAs-mediated miR-1-3p/Notch3/Smad3 axis may regulate sepsis-induced intestinal injury, and accordingly set out to perform a series of experiments to validate our hypothesis, aiming to uncover novel therapeutic targets against sepsis.

## Materials and methods

### Ethical approval

The current study was approved by the Animal Ethics Committee of the Shenzhen People’s Hospital. All experimental procedures were conducted in accordance with the National Institutes of Health, and extensive efforts were made to minimize the number and suffering of the included experimental animals.

### In silico analysis

First, downstream target genes of miR-1-3p were predicted using the Starbase database (http://starbase.sysu.edu.cn/index.php), the mirDIP database (http://ophid.utoronto.ca/mirDIP/index.jsp#r) and the miRDB database (http:// www.mirdb.org/). In addition, the sepsis-related gene expression profile GSE53007 was retrieved from the Gene Expression Omnibus database, which comprised of 4 normal samples and 4 sepsis samples. Subsequently, differential analysis was performed using the R language "limma" Package with *|logFoldChange|*> *0.5* and *p value* < *0.01* serving as the criteria to screen the differentially expressed genes. Afterwards, the predicted target gene candidates and the down-regulated genes from the GSE53007 dataset were intersected.

### Establishment of cecal ligation and perforation (CLP)-induced septic model

A total of 48 male C57BL/6J mice (aged 8–12 weeks) were purchased from the Animal Research Center of Jinan University. The obtained mice were raised in a specific-pathogen free animal laboratory in separate cages (humidity: 60–65%; temperature: 22–25 ℃), and given ad libitum access to food and water under 12-h light/dark cycles. Mice experimentation was commenced after acclimation for one week, and the health status of mice was observed prior to the experiment.

CLP was performed on the mice to establish septic models. Briefly, the anesthetized mice were fixed on a plate in the supine position. The cecum was then slightly pulled out through the midline incision of abdominal wall of the mice, and the feces of the upper cecum were squeezed to fill the end, followed by separation of the blood vessels on the mesenteric surface. Next, the midpoint between the cecal valve and the cecum was ligated using a sterile No. 4 silk thread, and the cecal wall was punctured using a 21G sterile needle at the middle point between the ligation site and the top of the cecum to cause perforation. The cecum was squeezed slightly to extrude a little content to confirm unobstructed perforation. Following laparotomy, the cecum of sham-operated mice was gently pulled-out without ligation, and subsequently pushed back into the abdominal cavity, which was then closed and sutured layer by layer.

### Animal treatment

Initially, the mice were subjected to sham-operation (n = 8) or CLP treatment (n = 24). After 24 h of CLP, CLP-treated mice were given gavage administration of omega-3 FAs (15 mg/mice) or gavage administration of both omega-3 FAs (15 mg/mice) and activin B (A1729-5UG, Sigma-Aldrich Chemical Company, St Louis, MO, USA) for two days, once per day. Omega-3 FAs was extracted from Omacor capsules (Pronova Biocare, Sandefjord, Norway) (each 1000 mg Omacor containing 460 mg EPA and 380 mg DHA). The body weight and survival status of the mice were monitored 3 days post-treatment. In addition, the expression patterns of miR-1-3p were determined using reverse transcription-quantitative polymerase chain reaction (RT-qPCR), and the activation of Notch3 and Smad signals in intestinal tissues was determined by means of a Western blot assay. The bacterial content in abdominal cavity and blood of mice was also detected, while the levels of pro-inflammatory factors in serum were examined by enzyme-linked immunosorbent assay (ELISA), and the levels of intestinal oxidative stress in mice were measured. Furthermore, the intestinal pathology was examined with the help of hematoxylin–eosin (HE) staining and transmission electron microscopy (TEM), while terminal deoxynucleotidyl transferase (TdT)-mediated deoxyuridine triphosphate (dUTP)-biotin nick end labeling (TUNEL) staining was adopted to examine the cell apoptosis in intestinal tissues, and immunohistochemistry was conducted to determine the expression patterns of cleaved caspase-3 (c-caspase-3).

### Detection of bacteria

The mice were anesthetized 72 h after CLP, and the abdominal skin of mice was gently sliced open to expose the peritoneum. Next, 3 mL of phosphate buffered saline (PBS) was slowly injected into the abdominal cavity through the peritoneum with a syringe. Next, the peritoneum was gently wiped with sterile cotton swabs, and the peritoneal lavage fluid was collected for subsequent bacterial culture. Afterwards, the hearts of the mice were completely exposed, and 200 μL of blood samples were collected and stored in sterile Eppendorf tubes for bacterial culture. Later, 50 μL of the collected peritoneal lavage fluid and 50 μL of blood samples were diluted 6 times using PBS, and evenly-smeared on a plate with trypsin soybean blood agar for culturing at 37 ℃ for 24 h. Finally, the number of formed colonies was counted and recorded.

### ELISA

The levels of tumor necrosis factor-α (TNF-α), interleukin 1β (IL-1β) and interleukin 6 (IL-6) in the serum and cell supernatant of mice were detected with the help of ELISA kits (Abcam Inc., Cambridge, MA, USA) according to the manufacturer’s instructions. In brief, 0.1 mL of cell supernatant was incubated in a 96-well plate at 37 ℃ for 1 h; blank wells were also set. After a rinse, 1 mL of freshly diluted enzyme-labeled antibody was added to each reaction well, and incubated at 37 ℃ for 0.5–1 h. Next, 0.1 mL of tetramethylbenzidine substrate solution was added to each reaction well, followed by incubation at 37 ℃ for 10–30 min. Afterwards, 0.05 mL of 2 M sulfuric acid was added to terminate the reaction. The optical density values of each well at 450 nm were measured, and the concentrations of TNF-α, IL-1β and IL-6 were calculated and recorded.

### Measurement of oxidative stress index

The level of reactive oxygen species (ROS) in tissues and cells was measured using the 2′,7′-dichlorofluorescein diacetate staining method with the help of ROS/Superoxide Detection Assay kits (Cell-based) (ab139476, Abcam). Firstly, the homogenate of colon tissues was extracted from the mice and diluted to a ratio of 1:10 in precooled PBS to obtain a tissue suspension with a tissue concentration of 5 mg/mL. Next, the homogenate was transferred to a 96-well plate with 10 μL per well, and incubated at room temperature for 5 min. Subsequently, 1 μM dichlorofluorescein diacetate was added to the homogenate, followed by incubation in conditions void of light at room temperature for 2 h. Following incubation, the cells were seeded in a 96-well plate for the cell experiment. After the intervention, the cells were incubated along with dichlorofluorescein diacetate in conditions void of light at room temperature for 20 min. The fluorescence intensity of free radicals was then quantitatively measured using a fluorescence spectrophotometer. The excitation and emission wavelengths were 488 and 525 nm, respectively, and the results were expressed by any fluorescence unit per milligram of protein.

Additionally, the levels of malondialdehyde (MDA) and superoxide dismutase (SOD) activity were measured using MDA detection kits (Colorimetric/Fluorometric) (ab118970, Abcam) and SOD detection kits (Colorimetric) (ab65354, Abcam), respectively. MDA in lipid peroxide degradation products was condensed with thiobarbituric acid to form a red product with the maximum absorption peak at a wavelength of 532 nm. In the presence of methionine and riboflavin, tetrazolium underwent a photochemical reduction reaction to form blue methylhydrazone, which exhibited the maximum light absorption at a wavelength of 560 nm. Meanwhile, SOD can inhibit the photochemical reduction of nitroblue tetrazolium, and the inhibition intensity is proportional to the enzyme activity in a certain range. According to the manufacturer’s instructions, the mice intestinal tissues were made into a 10% tissue homogenate. After adding the reagent, the mixture was whirled and evenly mixed. After 40 min of water bathing at 95 ℃, the mixture was centrifuged at 2800–3200 × g/min for 10 min. Next, the supernatant was collected for determination of MDA and SOD levels. For the cell experiment, the cells after intervention were made into a 10% homogenate, which was freeze-thawed thrice in a refrigerator at − 20 ℃. If the cells were not completely broken as observed under a microscope, they were further freeze-thawed twice and centrifuged at 3200 × g/minute for 15 min. Finally, the supernatant was collected from the homogenate for detection of SOD and MDA levels.

### HE staining

Paraffin-embedded distal colon sections of mice were immersed with xylene I and II (10 min for each), with anhydrous ethanol I and II (5 min for each), and then with gradient alcohol (95%, 90%, 80%, 70%, 5 min for each). Subsequently, the sections were rinsed with distilled water, dewaxed, dehydrated, and soaked in Harris hematoxylin for 3–8 min. After a wash under running water, the sections were then differentiated with 1% hydrochloric acid alcohol for several seconds, washed under running water, and treated with 0.6% ammonia water to return to blue coloration. Next, the sections were stained with eosin for 1–3 min, immersed with 95% alcohol I and II and in anhydrous ethanol I and II each for 5 min, dehydrated and cleared with xylene I and II each for 5 min. Afterwards, the sections were dried in air and sealed with neutral gum. Images were captured during microscopic examination for analyses of intestinal pathology.

### TEM

After 24 h of operation, colon samples from mice were collected, sectioned and fixed with 2.5% glutaraldehyde. Following rinsing with PBS (pH 7.0), the samples were re-fixed with 1% osmium tetroxide, and dehydrated with gradient ethanol and acetone. Next, the samples were paraffin-embedded, sliced and stained with uranium and lead (2% saturated uranyl acetate and lead citrate). Afterwards, images were then obtained using the Tecnai G2 TEM (FEI, Portland, Oregon, USA).

### TUNEL staining

Cell apoptosis in intestinal tissues was examined with the help of apoptosis detection kits (11684817910, Roche Diagnostics GmbH, Mannheim, Germany). The paraffin-embedded sections of mouse intestinal tissues were soaked with xylene twice, 5 min each, and then washed with gradient ethanol (100%, 95%, 90%, 80%, 70%), 3 min each. Subsequently, the sections were subjected to treatment with proteinase K working solution (20 μg/mL) at room temperature for 15–30 min. The treatment group was mixed with 50 μL of TdT + 450 μL fluorescein-labeled dUTP solution, while the negative control (NC) group was only added with 50 μL of fluorescein-labeled dUTP solution; the positive control group was treated with 100 μL DNase 1 at room temperature for 10–30 min (the following steps were the same as those in the treatment group). After the sections were dried, excess liquid around the sections was carefully sucked out with a filter paper, and 50 μL TUNEL reaction mixture was added to the samples (only 50 μL fluorescein labeled dUTP solution was added to the negative control group) for reaction in conditions void of light in a wet-box for 1 h at room temperature. The apoptotic cells were counted under a fluorescence microscope (excitation light wavelength: 450–500 nm; detection wavelength: 515–565 nm). Next, 50 μL converter-peroxidase was added to the samples, followed by 30-min reaction in conditions void of light in a wet-box. A total of 50–100 μL 3,3′-diaminobenzidine (DAB) substrate was added to the sections for 10-min reaction. After photographing, the samples were dyed with hematoxylin or methyl green, dehydrated with gradient alcohol, cleared with xylene and then sealed with neutral gum. Afterwards, the apoptotic cells (200–500 cells in total) were observed under a light microscope and photographed.

### Immunohistochemistry

The intestinal tissues of mice were fixed with 4% paraformaldehyde, paraffin-embedded, sliced into 4 μm thick sections, and dewaxed. Employing the streptavidin–biotin–peroxidase method, antigen repair was conducted. The sections were then rinsed with PBS and sealed with normal goat serum sealing solution. Normal tissues were used as NC during the experiment. Histostatin TMSP-9000 immunohistochemical staining kits (Zymed, San Francisco, CA, USA) were adopted to stain the sections. The sections were treated with the primary antibody cleaved-caspase-3 (ab2302, dilution ratio of 1 μg/mL) overnight at 4 ℃. The following day, the donkey anti-rabbit secondary antibody (ab6802, dilution ratio of 1:1000, donkey) was added drop-wise to the sections, followed by 30-min reaction at room temperature. Next, the sections were further incubated with horseradish-labeled working solution and subjected to DAB color development for 5–10 min. Following counterstaining with hematoxylin for 1 min, the sections were sealed with gum, baked, and photographed. Five representative high-power visual fields were selected for observation and counting, and those presenting with brown or yellow cytoplasm were regarded positive. The Image-Pro Plus version 6 software (Media Cybernetics, Silver Spring, Maryland, USA) were adopted to evaluate the integrated optical density of immunostaining samples.

### Cell treatment

Mouse intestinal epithelial MODE-K cells (BNCC338300) were procured from BeNa culture collection (BNCC, Beijing, China, http://www.bncc.org.cn). the cell bank of Chinese Academy of Sciences (Shanghai, China), and cultured in DMEM (high sugar) medium (PM150210, Procell Life Science&Technology Co., Ltd. Wuhan, China) containing 10% fetal bovine serum (Gibco) in a humidified incubator (Thermo Fisher Scientific, Rockford, IL, USA) at 37 ℃ with 5% CO_2_ in air. Next, the cells were detached with trypsin after reaching the logarithmic phase of growth, and then seeded in a 6-well plate, at a density of 1 × 10^5^ cells per well. After 24 h of culture, the cells were transfected according to the instructions of Lipofectamine 2000 (Invitrogen Inc., Carlsbad, CA, USA) with NC mimic, miR-1-3p mimic, short hairpin RNA (sh)-NC, sh-Notch3-1, and sh-Notch3-2. Subsequently, the efficiency of over-expressing miR-1-3p and that of silencing Notch3 were determined.

To further investigate the effect of omega-3 FAs on LPS-induced cell model, non-treated cells were used as control, while the remaining cells were treated with 1 μg/mL LPS (Standard lipopolysaccharide from *E. coli* 0111:B4 strain-toll-like receptor 4 ligand, Catalog # tlrl-eblps) for 24 h or treated with 1 μg/mL LPS for 24 h, and then treated with 1 μg of omega-3 FAs for 24 h. In order to identify the regulatory relationship between miR-1-3p and Notch3, 24 h prior to LPS treatment, the cells were transfected with NC inhibitor, miR-1-3p inhibitor, miR-1-3p inhibitor, miR-1-3p inhibitor + sh-NC, miR-1-3p inhibitor + sh-NC, or miR-1-3p inhibitor + sh-Notch3. Meanwhile, to examine the regulatory effect of Notch3 on the Smad pathway, 24 h prior to LPS treatment, the cells were transfected with over-expression (oe)-NC plasmid, oe-Notch3, or treated with activin (activin B, A1729-5UG, Sigma) in combination. Additionally, to assess the association between and omega-3 FAs and miR-1-3p/Notch3/Smad axis, the cells after 24 h of LPS treatment were subjected to treatment with omega-3 FAs (1 μL [1 mg/mL]; for 24 h) or omega-3 FAs (1 μL [1 mg/mL]; for 24 h) and activin (1 ng/mL; for 20 min) in combination.

### RT-qPCR

TRIzol kits (16096020 or AM1561, Thermo Fisher) were adopted to extract the total mRNA or miRNA content from tissues or cells. Subsequently, 5 μg of the RNA was reverse-transcribed into complementary DNA (cDNA) according to the instructions of RT-qPCR kits (RR047A, Takara, Kyoto, Japan), which was then subjected to real time qPCR with fast SYBR Green PCR kits (Applied Biosystems, Carlsbad, CA, USA) in an ABI PRISM 7300 RT-PCR system (Applied Biosystems). Three replicates were set in each well. With U6 serving as the internal reference for miR-1-3p and glyceraldehyde-3-phosphate dehydrogenase (GAPDH) for other genes, the 2^−ΔΔCT^ method was employed for calculating the relative expression of each target gene. The primer design is shown in Additional file 1: Table S1.

### Western blot assay

The cultured cells were collected by trypsin detachment, and then lysed with enhanced radio-immunoprecipitation assay lysis buffer containing a protease inhibitor (Boster, Wuhan, China). The protein concentration was determined using bicinchoninic acid protein assay kits (Boster). Subsequently, the proteins were separated using 10% sodium dodecyl sulfate-polyacrylamide gel electrophoresis gel, and transferred onto a polyvinylidene fluoride membrane which was sealed with 5% bovine serum albumin at room temperature for 1 h. Afterwards, the membranes were incubated overnight at 4 ℃ with diluted primary rabbit antibodies against caspase-3 (ab13847, dilution ratio of 1:500 μg/mL), c-caspase-3 (ab2302, dilution ratio of 1 μg/mL), Notch3 (ab23426, dilution ratio of 1 μg/mL) and phosphorylated (p)-Smad2/3 (ab272332, dilution ratio of 1:1000), Smad2/3 (ab217553, dilution ratio of 1: 500), Smad4 (ab40759, dilution ratio of 1:5000), and GAPDH antibody (internal reference, ab8245, dilution ratio of 1:5000, mouse). Next, the membranes were incubated with the horseradish peroxidase-labeled secondary antibodies (goat anti-rabbit, ab205718, dilution ratio of 1:10,000; goat anti-mouse, 1:10,000; ab205719) at room temperature for 1 h, followed by enhanced chemiluminescence development (Baoman Biotechnology, Shanghai, China). Afterwards, the gray value of each band was analyzed using the Image J gel image analysis software. All the aforementioned antibodies were purchased from Abcam.

### Flow cytometry

Cells in the culture dish were detached and collected using ethylenediaminetetracetic acid-free trypsin. Next, the cells in the supernatant were collected by centrifugation at 1600×*g* for 5 min, rinsed with PBS twice and then stained with 5 μL fluorescein isothiocyanate-Annexin V and 5 μL propidium iodide (KGA106, Keygen, Nanjing, China) for 15 min. Afterwards, apoptosis was examined with help of a flow cytometer (BD Biosciences, Franklin Lakes, NJ, USA).

### Dual-luciferase reporter gene assay

The biological prediction website www.microRNA.org, was utilized to predict the target gene of miR-1-3p and obtain their binding sequence. Subsequently, the Notch3 3′untranslated region (3′UTR) gene fragments were synthesized and introduced into the pMIR-reporter plasmid (Huayueyang Biotechnology, Beijing, China) using the endonuclease sites, *SpeI* and *Hind III*. The complementary sequence with mutation sites of the seed sequence were designed based on the wild type (WT)-Notch3 sequence. Following restriction endonuclease cleavage, the target fragments were inserted into the pMIR-reporter plasmid with T4 DNA ligase. The successfully sequenced luciferase reporter plasmids WT and mutant type (MUT) were then co-transfected with miR-1-3p mimic or mimic NC into MODE-K cells, respectively. After 48 h, the cells were collected and the protein content was extracted. The luciferase activity was measured using luciferase detection kits (K801-200, Biovision, Mountain View, CA, USA) and a lomax20/20 luminometer (Promega Corporation, Madison, WI, USA).

### Statistical analysis

Statistical analyses were performed using the SPSS 21.0 software (SPSS, IBM, Armonk, NY, USA). Measurement data were expressed as mean ± standard deviation from three independent experiments. Data between two groups were compared using unpaired *t*-tests. Data among multiple groups were compared by one-way analysis of variance (ANOVA) with Tukey's post-hoc test, while data at different time points were compared using repeated measures ANOVA with Tukey's post-hoc test. A value of *p* < 0.05 was considered statistically significant.

## Results

### Omega-3 FAs protected against sepsis-induced intestinal injury

Firstly, we established sepsis models in C57BL/6J mice by means of CLP. The mice were sham-operated or subjected to CLP treatment. On the 3^rd^ day, the weight of CLP-induced septic mice was measured and found to be decreased to 81%, while the death rate was as high as 75% (Additional file 2: Fig. S1A, B). Moreover, the bacterial content in CLP-induced septic mice was observed to be significantly higher than those in sham-operated mice (Additional file 2: Fig. S1C). In addition, the expression patterns of pro-inflammatory factors TNF-α, IL-1β and IL-6 were determined by ELISA, which were all found to be significantly higher in CLP-induced septic mice relative to sham-operated mice (Additional file 2: Fig. S1D). Meanwhile, determination of oxidative stress markers ROS, MDA and SOD in colon tissues revealed that the levels of ROS and MDA in CLP-induced septic mice were markedly increased, while those of SOD were significantly decreased, indicating that CLP-induced septic mice precipitated an obvious oxidative stress response (Additional file 2: Fig. S1E). In addition, the results of HE staining illustrated that, compared to sham-operated mice, CLP-induced septic mice exhibited more tissue destruction and inflammatory cell infiltration (Additional file 2: Fig. S1F). Furthermore, the intestinal epithelial cells of sham-operated mice were observed to be orderly-arranged, with visible tight junctions between the cells, while the intestinal epithelial cells in CLP-induced septic mice appeared to be swollen, with destroyed tight junctions between the cells (Additional file 2: Fig. S1G). Moreover, TUNEL staining demonstrated that relative to sham-operated mice, CLP-induced septic mice presented with increased cell apoptosis in intestine tissues (Additional file 2: Fig. S1H). Furthermore, the results of immunohistochemistry depicted that the expression of c-caspase-3 in CLP-induced septic mice was significantly higher than those in sham-operated mice (Additional file 2: Fig. S1I). Together, the abovementioned results indicated that the sepsis mouse models were successfully constructed, wherein CLP induced pronounced intestinal inflammation and oxidative stress injury. Subsequently, the expression patterns of pro-inflammatory factors and oxidative stress markers in the cells of each group were determined after omega-3 FAs intervention, which revealed that inflammation and oxidative stress response were markedly elevated by LPS intervention, while omega-3 treatment brought about a significant reduction in inflammation and oxidative stress response (Additional file 2: Fig. S1J, K). In addition, LPS significantly promoted cell apoptosis, while omega-3 FAs brought about the opposite trends (Additional file 2: Fig. S1L, M). Collectively, these findings indicated that the LPS-exposed intestinal epithelial cell model was successfully constructed, while omega-3 FAs could significantly inhibit the inflammation and oxidative stress injury of intestinal epithelial cells induced by LPS.

**Fig. 1:**
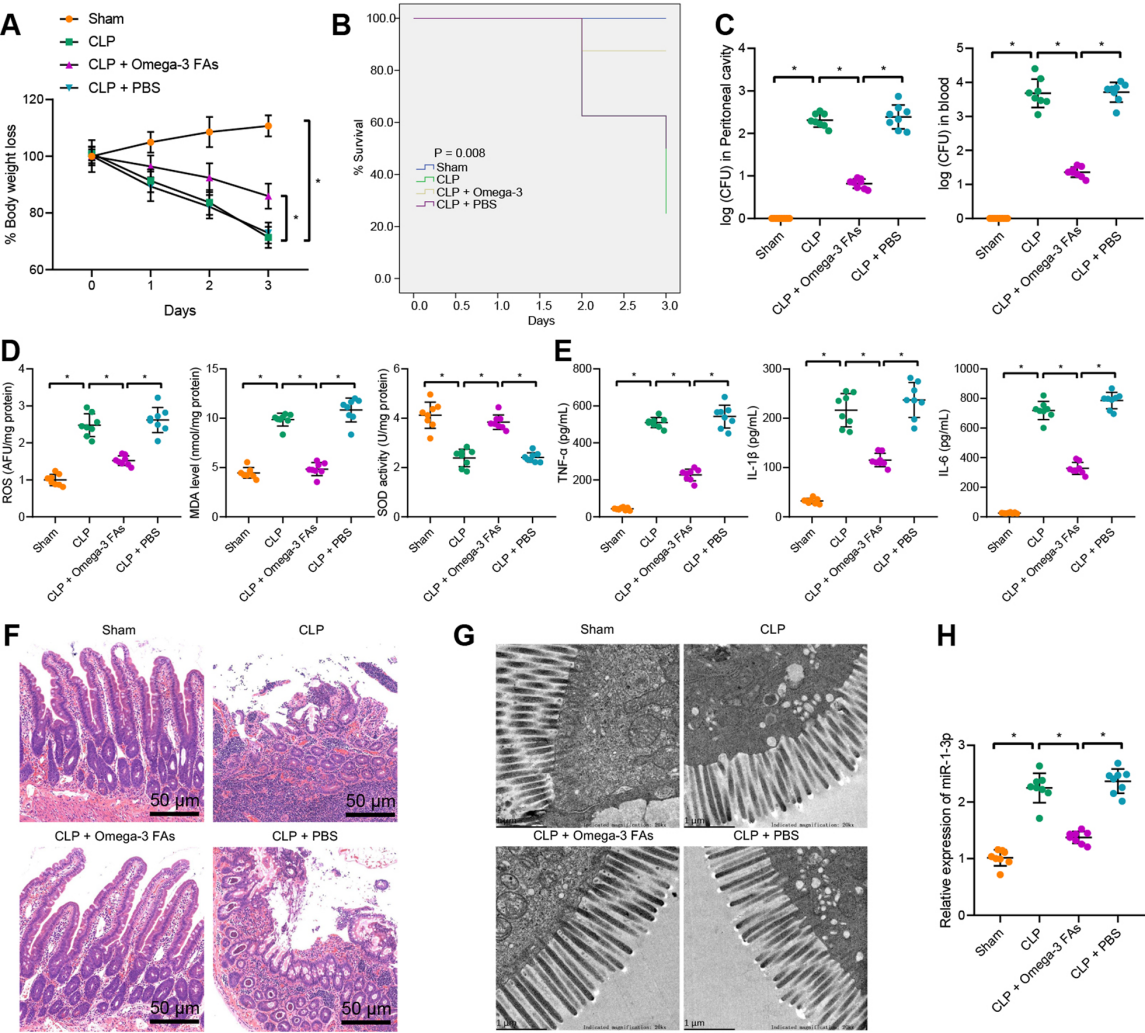
Omega-3 FAs alleviate inflammatory response and oxidative stress injury of intestinal epithelial cells induced by CLP. **A** Body weight of mice with different treatment within 3 days after sepsis induction. **B** Survival rate of mice with different treatment within 3 days after sepsis induction. **C** Bacterial content in peritoneal lavage fluid and blood of mice with different treatment. **D** The levels of ROS and MDA and the activity of SOD as detected by ROS, MDA and SOD detection kits. **E** The expression of TNF-α, IL-1β and IL-6 in serum of mice with different treatment as determined by ELISA. **F** Pathological changes of intestinal tissues of mice with different treatment as observed by HE staining. **G** The pathological changes of colonic mucosa as observed by TEM. **H** The miR-1-3p expression in intestinal tissues of mice determined by RT-qPCR; n = 8. The apoptosis of intestinal tissues as examined by TUNEL staining. *p < 0.05*.* Measurement data were expressed as mean ± standard deviation. One-way ANOVA with Tukey’s test was used for data analysis among multiple groups, and repeated measures ANOVA with Tukey's test for data analysis at different time points. Experiment was repeated independently three times

To further verify the inhibitory effect of omega-3 FAs on CLP-induced intestinal injury, we treated CLP-induced septic mice with omega-3 FAs or PBS (control). Three days post-operation, CLP-induced septic mice treated with omega-3 FAs presented with increased weight accompanied by reduced death rates (Fig. 1A, B), and lower bacterial content in peritoneal lavage fluid and serum (Fig. 1C) when compared to sham-operated mice or CLP-induced septic mice treated with PBS. In addition, lower ROS and MDA levels and higher SOD levels were observed in CLP-induced septic mice treated with omega-3 FAs, which is indicative of suppressed oxidative stress response (Fig. 1D). Additionally, the results of ELISA revealed down-regulated levels of TNF-α, IL-1β and IL-6 in the serum of CLP-induced septic mice treated with omega-3 FAs (Fig. 1E), while intestinal injury was observed to be alleviated following the addition of omega-3 FAs in CLP-induced septic mice, as illustrated by the results of HE staining and TEM (Fig. 1F, G). Furthermore, the presence of omega-3 FAs in CLP-induced septic mice led to low expressions of miR-1-3p, suggestive of an inverse relation between omega-3 FAs and miR-1-3p (Fig. 1H). Overall, these findings indicated that omega-3 FAs could significantly inhibit intestinal injury triggered by CLP.

### Omega-3 FAs ameliorated LPS-induced intestinal epithelial cell injury by down-regulating miR-1-3p

**Fig. 2:**
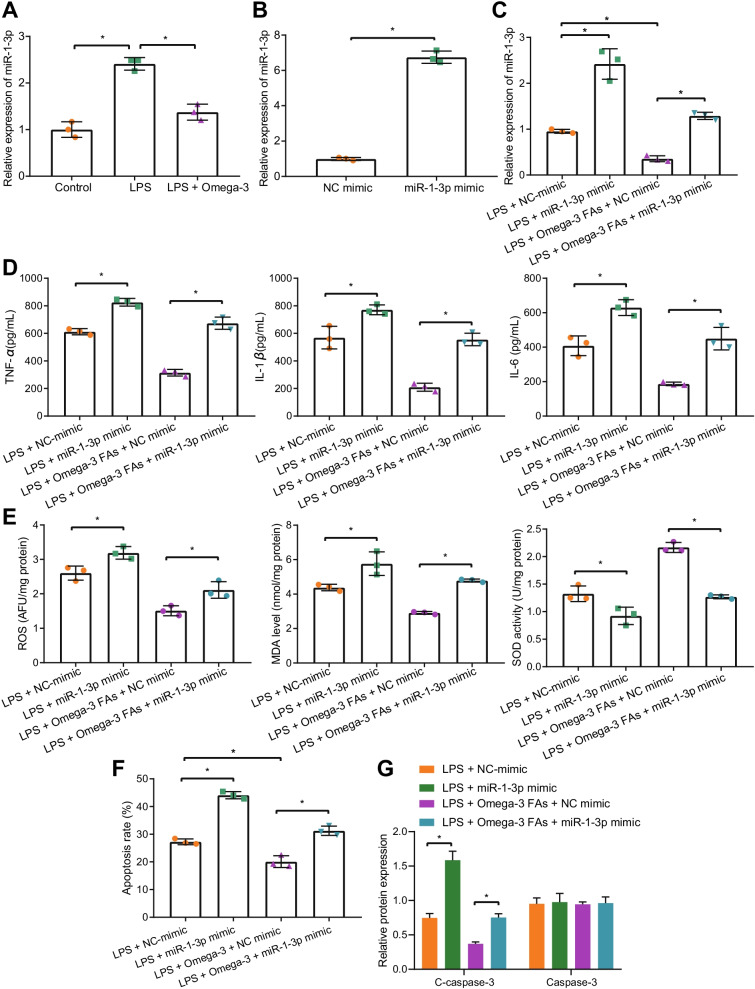
Omega-3 FAs ameliorate LPS-induced intestinal epithelial cell injury by downregulating miR-1-3p. **A** The expression of miR-1-3p in LPS-induced sepsis cell model as determined by RT-qPCR. **B** The transfection efficiency of miR-1-3p mimic as determined by RT-qPCR. **C** The expression of miR-1-3p in LPS-induced cells in response to miR-1-3p mimic or/and omega-3 FAs as determined by RT-qPCR. **D** The levels of TNF-α, IL-1β and IL-6 in the supernatant of LPS-induced cells in response to miR-1-3p mimic or/and omega-3 FAs as determined by ELISA. **E** The levels of ROS and MDA and the activity of SOD in LPS-induced cells in response to miR-1-3p mimic or/and omega-3 FAs as detected by ROS, MDA and SOD detection kits. **F** The apoptosis of LPS-induced cells in response to miR-1-3p mimic or/and omega-3 FAs as examined by flow cytometry. **G** The expression of cleaved-caspase-3 and caspase-3 in LPS-induced cells in response to miR-1-3p mimic or/and omega-3 FAs as detected by Western blot assay. *p < 0.05*.* Measurement data were expressed as mean ± standard deviation. Unpaired *t*-test was performed for the data analysis between two groups while one-way ANOVA with Tukey's test for data analysis among multiple groups. Experiment was repeated independently three times

Furthermore, we verified whether omega-3 FAs could play a therapeutic role in sepsis-induced intestinal injury by regulating the expression of miR-1-3p. First, the results of RT-qPCR demonstrated that miR-1-3p expression was significantly increased by LPS, while being decreased by omega-3 FAs, which suggested that omega-3 FAs significantly diminished the expression of miR-1-3p in sepsis (Fig. 2A). To further validate the abovementioned regulatory relationship, we transfected miR-1-3p mimic and NC mimic into mouse intestinal epithelial MODE-K cells, and verified the transfection efficiency using RT-qPCR (Fig. 2B). In addition, following omega-3 FA intervention in the LPS-exposed intestinal epithelial cell models, the expression patterns of miR-1-3p were determined by RT-qPCR after transfection with NC mimic and miR-1-3p mimic. The subsequent results revealed that the expression of miR-1-3p was markedly increased following transfection with miR-1-3p mimic in LPS-exposed intestinal epithelial cells treated with omega-3 FAs, while miR-1-3p expression levels were also increased in response to LPS + NC mimic compared to LPS + omega-3 FAs + NC mimic, while being lower than LPS + omega-3 FAs + miR-1-3p mimic (Fig. 2C). Moreover, omega-3 FAs brought about suppressed inflammatory response and oxidative stress, whereas additional over-expression of miR-1-3p led to augmented inflammatory response and oxidative stress (Fig. 2D, E). Meanwhile, the results of flow cytometry and Western blot assay showed that the addition of omega-3 FAs inhibited cell apoptosis, whereas over-expression of miR-1-3p brought about the opposite trends (Fig. 2F, G, Additional file 3: Fig. S2A, and Additional file 4: Fig. S3A). Collectively, these findings indicated that omega-3 FAs ameliorated LPS-induced inflammation and oxidative stress injury of intestinal epithelial cells by inhibiting the expression of miR-1-3p.

### miR-1-3p down-regulated Notch3 and aggravated LPS-induced intestinal epithelial cell injury

**Fig. 3:**
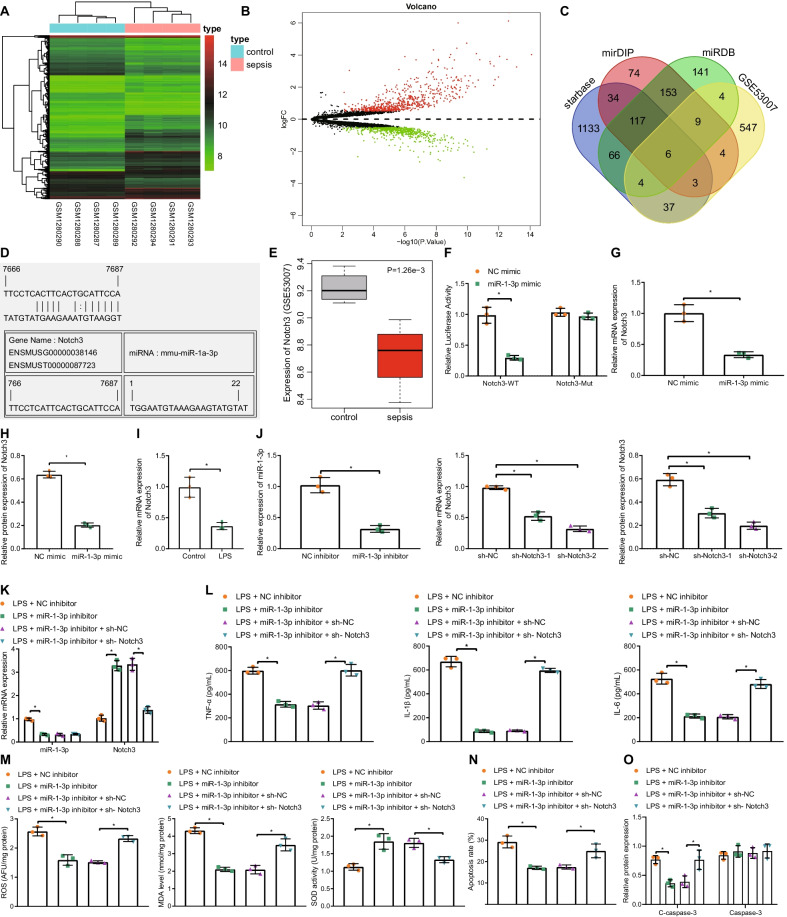
miR-1-3p down-regulates Notch3 and promotes LPS-induced intestinal epithelial cell injury. **A** The heatmap of differentially expressed genes in sepsis obtained from the profile GSE53007. **B** Volcano plot of differentially expressed genes in sepsis. **C** The intersection of target genes of miR-1-3p predicted by starbase, mirDIP, and miRDB databases and down-regulated genes in sepsis from GSE53007. **D** The miR-1-3p binding sites in Notch3. **E** The expression of Notch3 in normal and sepsis samples from GSE53007. **F** The relationship between miR-1-3p and Notch3 as examined by dual-luciferase reporter gene assay. **G** The effect of miR-1-3p on Notch3 mRNA expression as determined by RT-qPCR. **H** The effect of miR-1-3p on Notch3 protein expression as determined by Western blot assay. **I** The expression of Notch3 in LPS-induced cells as determined by RT-qPCR. **J** The silencing effect of miR-1-3p and sh-Notch3 as examined by RT-qPCR and Western blot assay. **K** The expression of miR-1-3p and Notch3 in LPS-induced cells treated with miR-1-3p inhibitor or miR-1-3p inhibitor + sh-Notch3 as determined by RT-qPCR. **L** The levels of TNF-α, IL-1β and IL-6 in supernatant of LPS-induced cells treated with miR-1-3p inhibitor or miR-1-3p inhibitor + sh-Notch3 as determined by ELISA. **M** Determination of ROS and MDA levels and SOD activity in LPS-induced cells treated with miR-1-3p inhibitor or miR-1-3p inhibitor + sh-Notch3. **N** The apoptosis of LPS-induced cells treated with miR-1-3p inhibitor or miR-1-3p inhibitor + sh-Notch3 as examined by TUNEL staining. **O** The expression of cleaved-caspase-3 and caspase-3 in LPS-induced cells treated with miR-1-3p inhibitor or miR-1-3p inhibitor + sh-Notch3 as determined by Western blot assay. *p < 0.05*.* Measurement data were expressed as mean ± standard deviation. Unpaired *t*-test was performed for the data analysis between two groups while one-way ANOVA with Tukey's test for data analysis among multiple groups. Experiment was repeated independently three times

Analyses of the expression profile GSE53007 yielded a total of 651 up-regulated genes and 614 down-regulated genes in sepsis samples (Fig. 3A, B). Subsequently, intersection of the top 1400 target genes obtained from the Starbase database, the top 400 target genes obtained from the mirDIP database, the top 500 target genes obtained from the miRDB database and the 614 down-regulated genes in GSE53007 was obtained (Fig. 3C, D), which revealed that the CORO1C, BCL7A, ANXA2, ETS1, MMD, and Notch3 genes at the intersection. Among those genes, Notch3 was found to be significantly down-regulated in sepsis as evidenced by the GSE53007 dataset (Fig. 3E). A dual luciferase reporter gene assay was then performed to verify that Notch3 was indeed the target of miR-1-3p in mouse intestinal epithelial MODE-K cells, and the experimental results demonstrated that compared with NC mimic, miR-1-3p mimic failed to notably alter the luciferase activity of MUT Notch3, but significantly down-regulated that of WT Notch3 (Fig. 3F). In addition, the results of RT-qPCR and Western blot assay illustrated that miR-1-3p mimic transfection brought about down-regulated expressions of Notch3 (Fig. 3G, H, Additional file 3: Fig. S2B). Together, these findings suggested that miR-1-3p specifically-inhibited the expression of Notch3. Moreover, the expression of Notch3 in cells treated with LPS was significantly lower than those in control cells (Fig. 3I).

To further investigate the effect of miR-1-3p on the intestinal epithelial cell injury, MODE-K cells were transfected with NC inhibitor, miR-1-3p inhibitor, sh-NC, sh-Notch3-1 or sh-Notch3-2. RT-qPCR and Western blot assay were subsequently performed to measure the transfection efficiency of miR-1-3p inhibitor and sh-Notch3, which revealed that miR-1-3p inhibitor significantly diminished the expression of miR-1-3p, such that sh-Notch3-2 exhibited more pronounced silencing efficiency than sh-Notch3-1, and was thus selected for further experimentation (Fig. 3J, Additional file 3: Fig. S2C). LPS-exposed intestinal epithelial cells were then transfected with miR-1-3p inhibitor and sh-Notch3, respectively, and the expression patterns of miR-1-3p and Notch3 were determined using RT-qPCR. It was found that miR-1-3p inhibitor significantly reduced the miR-1-3p expression, while increasing that of Notch3; meanwhile, sh-Notch3 brought about no significant changes in miR-1-3p expression, but decreased that of Notch3 (Fig. 3K). Further detection of pro-inflammatory factors and oxidative stress marker expression patterns revealed that miR-1-3p inhibitor brought about markedly inhibited LPS-induced cell inflammation and oxidative stress responses, while sh-Notch3 exhibited the opposite trends (Fig. 3L, M). As shown by the results of flow cytometry and Western blot assay, apoptosis induced by LPS was significantly reduced by miR-1-3p inhibition, whereas this effect could be significantly countered by sh-Notch3 (Fig. 3N, O, and Additional file 4: Fig. S3B). Altogether, these results suggested that miR-1-3p promoted LPS-induced inflammation and oxidative stress injury in intestinal epithelial cells by targeting Notch3.

### Notch3 inhibited the Smad signaling and attenuated LPS-induced intestinal epithelial cell injury

**Fig. 4:**
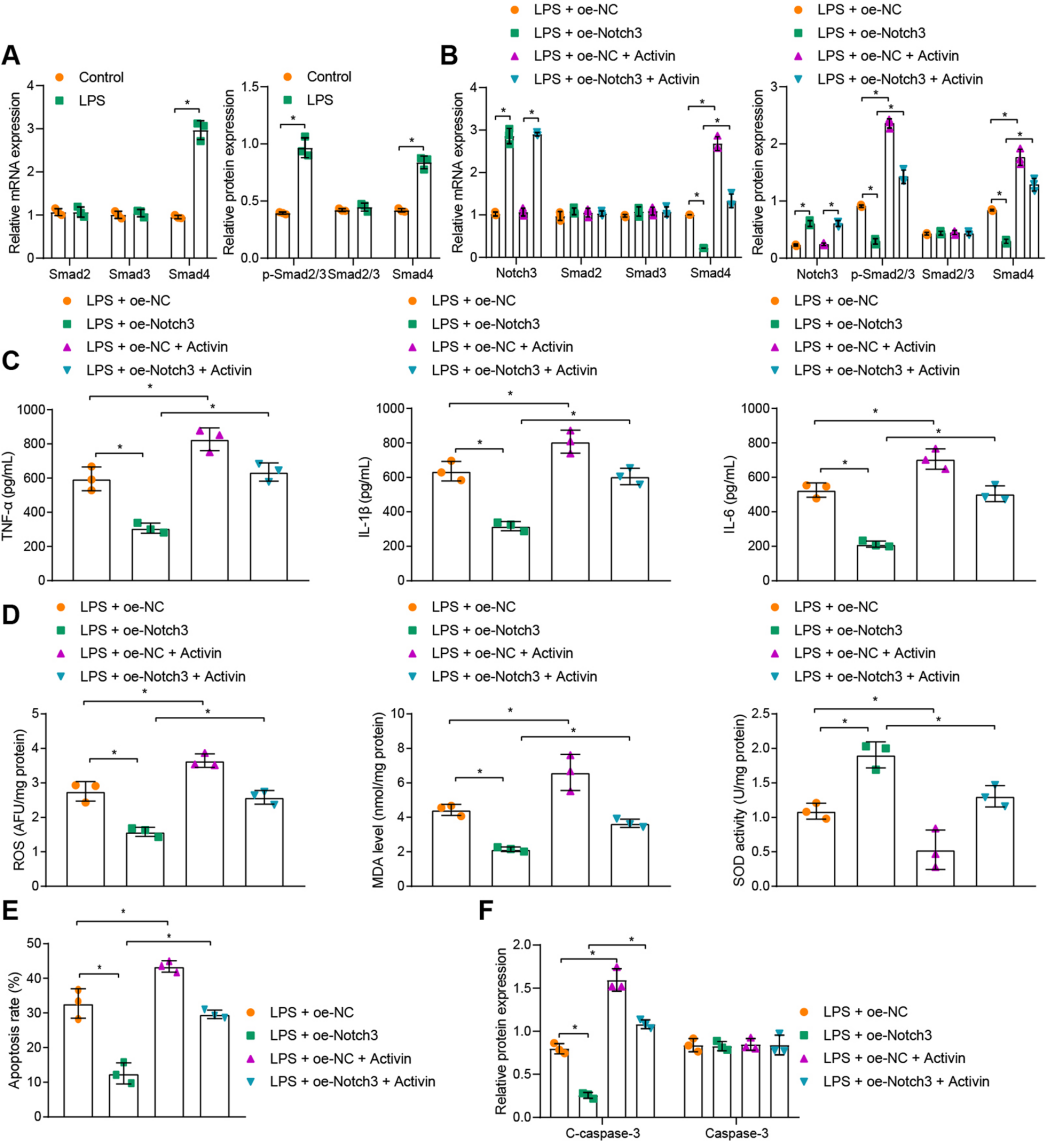
Notch3 inhibits the Smad signaling and attenuates LPS-induced intestinal epithelial cell injury. **A** The activation of Smad in the sepsis cell model as measured by Western blot assay. **B** The activation of Notch3 and Smad in LPS-induced cells treated with oe-Notch3 or oe-Notch3 + activin as measured by Western blot assay. **C** The levels of TNF-α, IL-1β and IL-6 in supernatant of LPS-induced cells treated with oe-Notch3 or oe-Notch3 + activin as determined by ELISA. **D** Determination of ROS and MDA levels and SOD activity in LPS-induced cells treated with oe-Notch3 or oe-Notch3 + activin. **E** The apoptosis of LPS-induced cells treated with oe-Notch3 or oe-Notch3 + activin as examined by TUNEL staining. **F** The expression of cleaved-caspase-3 and caspase-3 in LPS-induced cells treated with oe-Notch3 or oe-Notch3 + activin as determined by Western blot assay. *p < 0.05*.* Measurement data were expressed as mean ± standard deviation. Unpaired *t*-test was performed for the data analysis between two groups while one-way ANOVA with Tukey's test for data analysis among multiple groups. Experiment was repeated independently three times

Furthermore, we explored whether Notch3 regulates sepsis-induced intestinal injury via inhibition of Smad signaling. First, a Western blot assay was performed to examine the activation of Smad signaling in the LPS-induced cell models, which revealed that the extent of p-Smad2/3 and Smad4 expression were significantly higher in intestinal epithelial cells exposed to LPS than those in the control cells (Fig. 4A, Additional file 3: Fig. S2D), which indicated that Smad signaling was indeed activated in intestinal cells in the context of LPS-induced injury. Activins are well-known as members of the TGF superfamily as a Smad signaling activator (Tang et al. 2019). MODE-K cells exposed to LPS were further treated with oe-NC, oe-Notch3, oe-NC + activin or oe-Notch3 + activin in combination, and the results of Western blot assay demonstrated that oe-Notch3 brought about an increase in the expression of Notch3, while decreasing the relative expression of p-Smad2/3 and Smad4, whereas activin treatment exerted no significant changes in Notch3 expression, but increased the extent of p-Smad2/3 and the Smad4 expression. Compared to oe-Notch3, oe-Notch3 + activin treatment was found to lead to no significant changes in Notch3 expression, while increasing the relative expression of p-Smad2/3 and the Smad4 expression (Fig. 4B). Moreover, we observed that LPS-induced inflammation and oxidative stress response in the intestinal epithelial cells were significantly reduced by oe-Notch3, whereas activin treatment led to the opposite trends (Fig. 4C, D). Flow cytometry and Western blot assay further demonstrated that apoptosis induced by LPS was significantly reduced upon oe-Notch3, while being markedly increased as a result of activin treatment (Fig. 4E, F, Additional file 3: Fig. S2E, and Additional file 4: Fig. S3C). Together, these findings suggested that Notch3 inhibited the activation of Smad signal, thereby reducing the inflammation and oxidative stress injury of intestinal epithelial cells induced by LPS.

### Omega-3 FAs attenuated LPS-induced intestinal epithelial cell injury through regulation of the miR-1-3p/Notch3/Smad axis

**Fig. 5:**
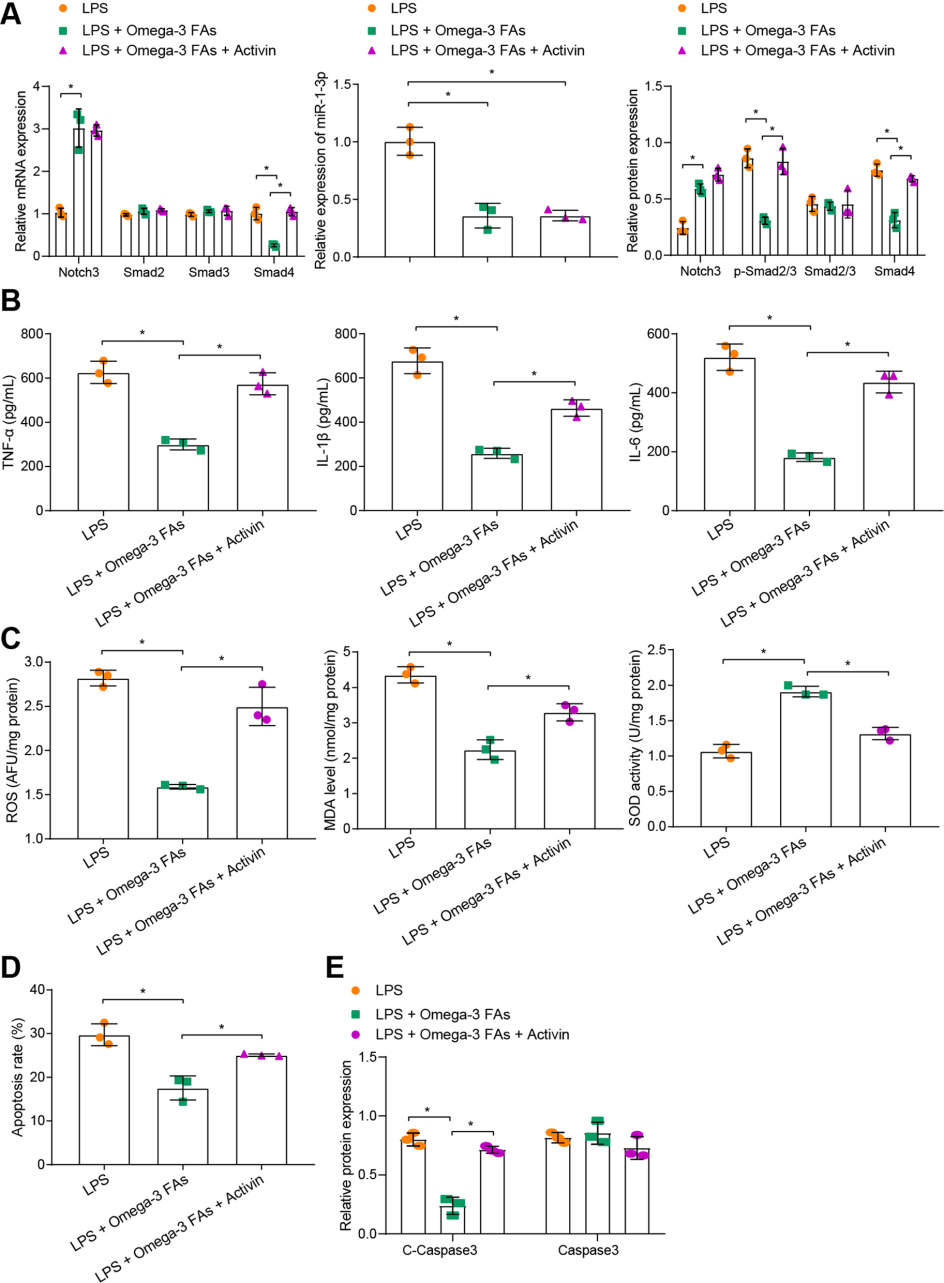
Omega-3 FAs mediate the miR-1-3p/Notch3/Smad axis to attenuate LPS-induced intestinal epithelial cell injury. **A** The expression of miR-1-3p as determined by RT-qPCR and the activation of Notch3 and Smad in LPS-induced cells treated with omega-3 FAs or omega-3 FAs + activin as measured by Western blot assay. **B** The levels of TNF-α, IL-1β and IL-6 in supernatant of LPS-induced cells treated with omega-3 FAs or omega-3 FAs + activin as determined by ELISA. **C** Determination of ROS and MDA levels and SOD activity in LPS-induced cells treated with omega-3 FAs or omega-3 FAs + activin. **D** The apoptosis of LPS-induced cells treated with omega-3 FAs or omega-3 FAs + activin as examined by TUNEL staining. **E** The expression of cleaved-caspase-3 and caspase-3 in LPS-induced cells treated with omega-3 FAs or omega-3 FAs + activin as determined by Western blot assay. *p < 0.05*.* Measurement data were expressed as mean ± standard deviation. Unpaired *t*-test was performed for the data analysis between two groups. Experiment was repeated independently three times

In order to further validate that omega-3 FAs could reduce the inflammation and apoptosis of intestinal epithelial cells induced by LPS by regulating the miR-1-3p/Notch3/Smad axis, we treated intestinal epithelial cells (MODE-K) with omega-3 FAs or omega-3 FAs + activin in combination after LPS intervention. As shown by the results of RT-qPCR and Western blot assay, individual omega-3 FAs treatment down-regulated the expressions of miR-1-3p, p-Smad2/3 and Smad4, while up-regulating those of Notch3, whereas omega-3 FAs + activin treatment did not alter the expressions of miR-1-3p and Notch3, but markedly increased the p-Smad2/3 extent and Smad4 expression in LPS-exposed MODE-K cells (Fig. 5A, Additional file 3: Fig. S2F). Meanwhile, individual omega-3 FAs treatment brought about marked suppression of inflammation and oxidative stress response, while being aggravated by omega-3 FAs + activin treatment in LPS-exposed MODE-K cells (Fig. 5B, C). Furthermore, flow cytometry and Western blot assay illustrated that individual omega-3 Fas treatment exerted an inhibitory effect on cell apoptosis, whereas omega-3 Fas + activin treatment resulted in the opposite trends (Fig. 5D, E, and Additional file 4: Fig. S3D). Altogether, these results indicated that omega-3 FAs were capable of alleviating LPS-induced inflammation and oxidative stress injury of intestinal epithelial cells through regulation of the miR-1-3p/Notch3/Smad axis.

### Omega-3 FAs alleviated sepsis-induced intestinal injury via mediation of the miR-1-3p/Notch3/Smad axis

**Fig. 6:**
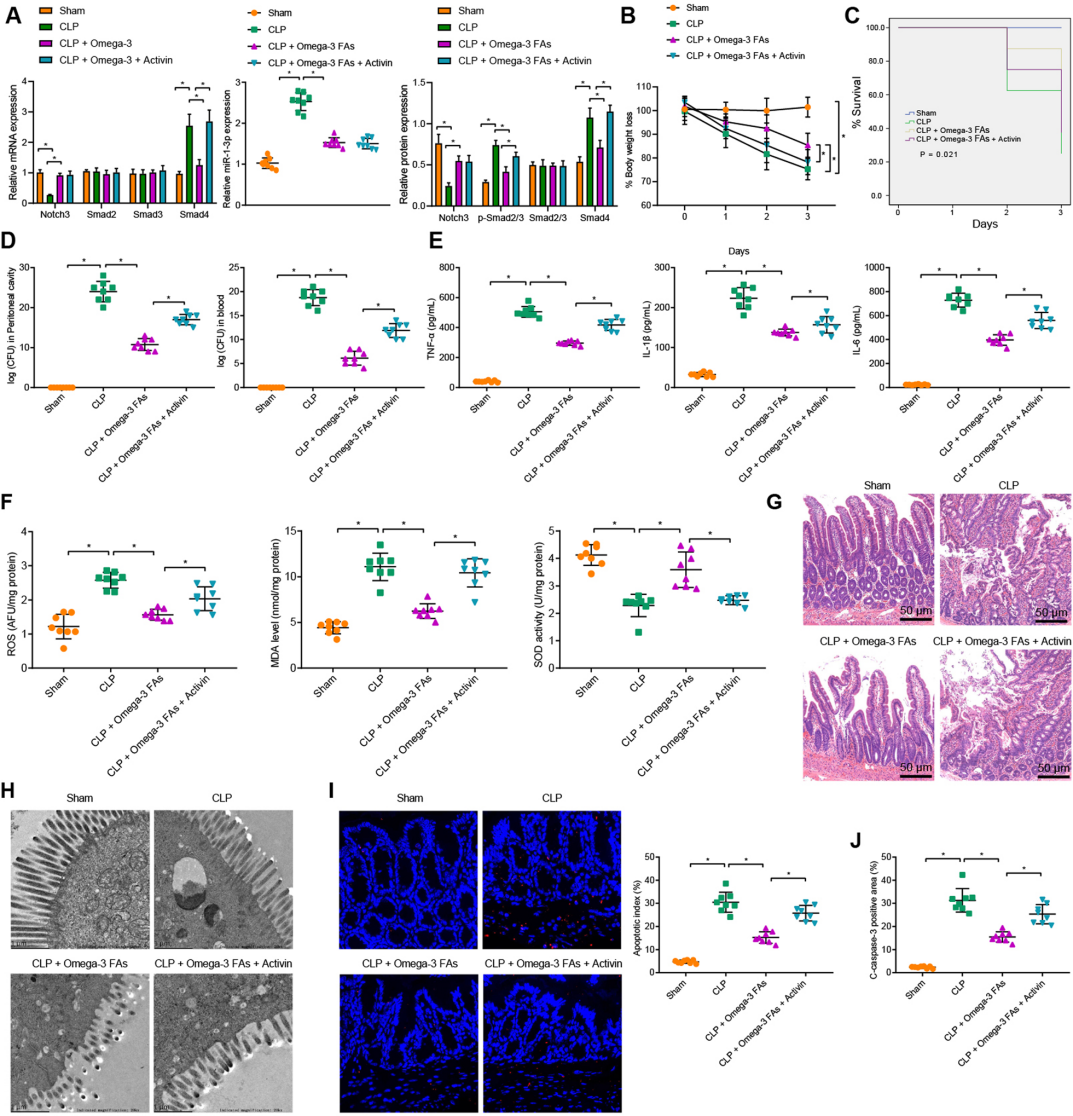
Omega-3 FAs mediate the miR-1-3p/Notch3/Smad signal axis to alleviate sepsis-induced intestinal injury. **A** The expression of miR-1-3p and the activation of Notch3 and Smad in intestinal tissues of mice with different treatment as measured by RT-qPCR and Western blot assay. **B** Body weight of mice with different treatment within 3 days after sepsis induction. **C** Survival rate of mice with different treatment within 3 days after sepsis induction. **D** Bacterial content in peritoneal lavage fluid and blood of mice with different treatment. **E** The expression of TNF-α, IL-1β and IL-6 in serum of mice with different treatment as determined by ELISA. **F** Determination of ROS and MDA levels and SOD activity in mice with different treatment. **G** Pathological changes of intestinal tissues of mice with different treatment as observed by HE staining. **H** The pathological changes of colonic mucosa as observed by TEM. **I** The apoptosis of intestinal tissues as examined by TUNEL staining. **J** The expression of cleaved-caspase-3 and caspase-3 as determined by immunohistochemistry. n = 8. *p < 0.05*.* Measurement data were expressed as mean ± standard deviation. Unpaired *t*-test was performed for the data analysis between two groups while one-way ANOVA with Tukey's test for data analysis among multiple groups, and repeated measures ANOVA with Tukey's test for data analysis at different time points. Experiment was repeated independently three times

Lastly, in order to elucidate the underlying mechanism by which omega-3 FAs attenuate sepsis-induced intestinal injury, we established CLP-induced septic mouse models, which were further treated with omega-3 FAs or omega-3 FAs + activin in combination. As illustrated by the results of RT-qPCR and Western blot assay, compared with sham-operated mice, CLP-induced septic mice presented with increased miR-1-3p expression, p-Smad2/3 extent and Smad4 expression, while the Notch3 expression was decreased. Meanwhile, treatment with omega-3 FAs brought about decreased miR-1-3p expression, p-Smad2/3 extent and Smad4 expression in CLP-induced septic mice, while that of Notch-3 was increased, and these effects could be neutralized by activin treatment (Fig. 6A, Additional file 3: Fig. S2G). In addition, the body weight and survival rate of mice were measured 3 days after operation, which revealed that on the 3^rd^ day, relative to individual omega-3 FAs treatment, omega-3 FAs + activin treatment significantly decreased the body weight, and increased the mortality rate in CLP-induced septic mice (Fig. 6B, C). Moreover, the bacterial contents in peritoneal lavage fluid and serum were observed to be significantly higher in CLP-induced septic mice treated with omega-3 FAs + activin compared to those treated with omega-3 FAs (Fig. 6D). The results of ELISA further demonstrated that the levels of TNF-α, IL-1β and IL-6 in CLP-induced septic mice were all markedly increased in response to omega-3 FAs + activin treatment compared to those following omega-3 FAs treatment (Fig. 6E). Furthermore, compared with CLP-induced septic mice treated with omega-3 FAs, those treated with omega-3 FAs + activin presented with increased levels of ROS and MDA, and decreased SOD levels, all suggestive of enhanced oxidative stress response (Fig. 6F). HE staining and TEM illustrated that activin aggravated the intestinal injury and attenuated the therapeutic effect of omega-3 FAs (Fig. 6G, H). Additionally, the results of TUNEL staining depicted that cell apoptosis in intestinal tissues was augmented following activin treatment, which inhibited the therapeutic effect of omega-3 FAs (Fig. 6I). As shown in immunohistochemistry, compared with individual omega-3 FAs treatment, omega-3 FAs + activin treatment brought about increased expressions of cleaved-caspase-3 in CLP-induced septic mice (Fig. 6J). Overall, these findings suggested that omega-3 FAs modulated the miR-1-3p/Notch3/Smad axis, thereby reducing intestinal epithelial inflammation and oxidative stress injury caused by sepsis.

## Discussion

Sepsis brings about a large proportion of mortality and morbidity all over the globe, with inflammation and oxidative stress playing crucial roles (Aydin et al. 2020). Meanwhile, the hard work of our peers has highlighted the involvement of intestinal injury in the pathophysiology of sepsis (Zou et al. 2019). Therefore, the current study set out to explore the underlying mechanism by which omega-3 FAs could affect sepsis-induced intestinal injury, and the obtained findings uncovered that omega-3 FAs alleviated sepsis-induced intestinal injury by down-regulating miR-1-3p, elevating Notch3 and blocking the activation of the Smad pathway.

Initial findings obtained in our study first illustrated that omega-3 FAs exerted an inhibitory effect on sepsis-induced intestinal injury in both animal and cell models, as evidenced by reduced levels of inflammation and diminished oxidative stress injury. Interestingly, a prior study suggested that omega-3 fish oil is capable of diminishing mortality in septic patients with Grade III acute gastrointestinal injury (Chen et al. 2017). In addition, omega-3 polyunsaturated fatty acids are known to improve metabolic processes as well as alleviate inflammation-triggered organ dysfunction in patients with sepsis, thus serving as a nutritional therapeutic against sepsis (De Waele et al. 2020). More recently, an omega-3 polyunsaturated fatty acid-derived metabolite, Resolvin E1 was documented to inhibit inflammatory responses and apoptotic damage in sepsis-induced cardiomyopathy, which is in accordance with our discovery (Zhang et al. 2020). Further in line with our findings, Sun et al*.* demonstrated that omega-3 fatty acids also possess the ability to ameliorate irradiation-induced gastrointestinal injury in BALB/c mouse models by diminishing TNF-α, IL-6 and MDA levels, as well as increasing SOD activity (Sun et al. 2014). Altogether, these findings and data make it plausible to suggest that omega-3 FAs exhibit a protective effect against sepsis.

Subsequent mechanistic investigation in our study further revealed that omega-3 FAs inhibited the expression of miR-1-3p to ameliorate LPS-induced intestinal epithelial cell injury. Moreover, we also uncovered a regulatory relationship between omega-3 FAs and miR-1-3p, whereas miR-1-3p targeted and down-regulated Notch3 to aggravate LPS-induced intestinal epithelial cell injury. The investigation by Han et al*.* recently demonstrated miR-1-5p can deteriorate LPS-induced mitochondrial damage as well as inhibit apoptosis in septic mice, which is very similar to our findings (Han et al. 2020). Further in accordance with our data, another study illustrated that miR-1 targeted Notch3 in H9c2 cells under oxidative stress, resulting in aggravation of hypoxia-induced myocardial ischemia injury (Xu et al. 2020). Meanwhile, miR-1 is also known to down-regulate Notch3 by directly-binding to its 3′UTR region in colorectal tumor cells (Furukawa et al. 2013). It is also noteworthy that Chen et al*.* illustrated the canonical Notch signaling was capable of suppressing the apoptosis of intestinal epithelial cells post-intestinal ischemia/reperfusion injury (Chen et al. 2014). As such we are the first to illustrate that omega-3 FAs impair miR-1-3p-mediated inhibition of Notch3 to alleviate sepsis-induced intestinal injury.

Additionally, we further uncovered that Notch3 inhibited the Smad pathway to alleviate LPS-induced intestinal epithelial cell injury. This is particularly noteworthy as Smad3-related signaling pathways are widely-known to participate in the development of sepsis or intestinal injury. On the other hand, inactivation of the TGF-β1/Smad3 pathway caused by curcumin was previously demonstrated to aid the alleviation of sepsis-induced acute lung injury in rat models (Xu et al. 2013). Meanwhile, TGF-β possesses the ability to modulate LPS-stimulated macrophage M2 polarization to enhance inflammatory response, ensuing aggravation of sepsis progression (Liu et al. 2019). In addition, a prior study suggested that over-expression of TGF-β is capable of attenuating organ dysfunction in mice with sepsis induced by CLP (Liu et al. 2020). Conversely, blockade of TGF-β contributes to septic death (Yeh et al. 2002), which suggests a protective effect of TGF-β in sepsis. Furthermore, in vivo experimentation in our study illustrated that omega-3 FAs impaired the miR-1-3p-mediated inhibition of Notch3 and disrupted the Smad pathway to attenuate sepsis-induced intestinal injury with the help of murine models. On a separate note, the study performed by Zhang et al*.* further suggested that activation of the TGFBR2/Smad pathway was responsible for LPS-induced sepsis, resulting in increased levels of IL-2 and TNF-α and reduced overall survival of mice with sepsis (Zhang et al. 2019). In addition, suppression of the TGF-β1/Smad/connective tissue growth factor pathway by pirfenidone was further indicated to prevent radiation-induced intestinal fibrosis in rat models by means of repressing fibroblast proliferation as well as differentiation (Sun et al. 2018). In lieu of these findings, it would be reasonable to infer the involvement of TGF-β1/Smad pathway in sepsis progression. Additionally, various studies have explored the regulatory effect of Notch pathway on Smad3. For instance, Notch1 was shown to inhibit the TGF-β/Smad3 signaling to disrupt cardiac fibroblast-myofibroblast transition (Sassoli et al. 2013); whereas, Notch3 is known to suppress the activation of Smad3 signal in cardiac fibrosis (Zhang et al. 2016). Altogether, these data and evidences are highly-suggestive of the critical role of Smad pathway inhibition Notch3 in sepsis-induced intestinal injury.

## Conclusions

**Fig. 7:**
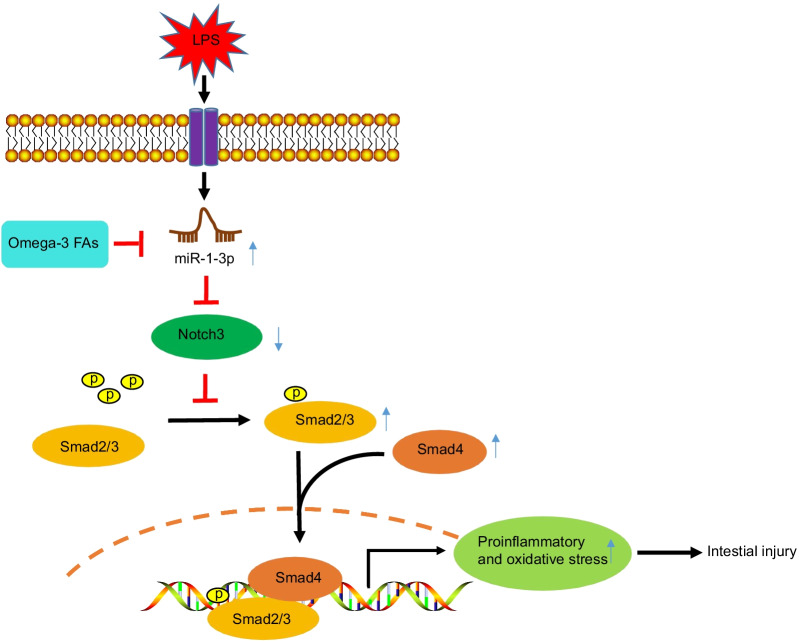
Omega-3 FAs-mediated miR-1-3p/Notch3/Smad axis affects sepsis-induced intestinal injury

In summary, findings obtained in our study indicate that omega-3 FAs are capable of inactivating the miR-1-3p/Notch3/Smad axis, and consequently alleviating sepsis-induced intestinal injury (Fig. 7). Our discoveries provide a deeper and better understanding of the protective mechanism of the miR-1-3p/Notch3/Smad axis in sepsis-induced intestinal injury. Nevertheless, it is imperative to explore and validate more specific mechanisms to improve the quality of life of patients affected by sepsis.

## Supplementary Information

**Additional file 1: Table S1.** Primer sequences for RT-qPCR. Note: RT-qPCR, reverse transcription-quantitative polymerase chain reaction; miR-1-3p, microRNA-1-3p; GAPDH, glyceraldehyde-3-phosphate dehydrogenase; F, forward; R, reverse.

**Additional file 2: Figure S1.** Omega-3 FAs inhibit sepsis-induced intestinal injury both in vitro and in vivo. A, Body weight of mice with different treatment within 3 days after sepsis induction. B, Survival rate of mice with different treatment within 3 days after sepsis induction. C, Bacterial content in peritoneal lavage fluid and blood of mice with different treatment. D, The expression of TNF-α, IL-1β and IL-6 in serum of mice with different treatment as determined by ELISA. E, Determination of ROS and MDA levels and SOD activity in mice with different treatment. F, Pathological changes of intestinal tissues of mice with different treatment as observed by HE staining (200 ×). G, The pathological changes of colonic mucosa as observed by TEM (500 nm). H, The apoptosis of intestinal tissues as examined by TUNEL staining. I, The expression of cleaved-caspase-3 and caspase-3 as determined by immunohistochemistry. J, The levels of TNF-α, IL-1β and IL-6 in the supernatant of cells with different treatment as determined by ELISA. K, The levels of ROS and MDA and the activity of SOD as detected by ROS, MDA and SOD detection kits. L, The apoptosis of cells with different treatment as examined by flow cytometry. M, The expression of cleaved-caspase-3 and caspase-3 in cells with different treatment as detected by Western blot assay. n = 8 in the in vivo experiment and n = 3 in the in vitro experiment. *p < 0.05. Measurement data were expressed as mean ± standard deviation. Unpaired t-test was performed for the data analysis between two groups while one-way ANOVA with Tukey's test for data analysis among multiple groups, and repeated measures ANOVA with Tukey's test for data analysis at different time points. Experiment was repeated independently three times.

**Additional file 3: Figure S2.** A, Representative protein bands of Figure 2G. B, Representative protein bands of Figure 3H. C, Representative protein bands of Figure 3J. D, Representative protein bands of Figure 4A. E, Representative protein bands of Figure 4B. F, Representative protein bands of Figure 5A. G, Representative protein bands of Figure 6A.

**Additional file 4: Figure S3.** A, Representative image of Figure 2F. B, Representative image of Figure 3N. C, Representative image of Figure 4E. D, Representative image of Figure 5D.

## Data Availability

The datasets generated and/or analysed during the current study are available from the corresponding author on reasonable request.

## Abbreviations

CLP: Cecal ligation and perforation
LPS: Lipopolysaccharide
miRs: MicroRNAs
ELISA: Enzyme-linked immunosorbent assay
HE: Hematoxylin–eosin
TEM: Transmission electron microscopy
TUNEL: Transferase-mediated dUTP-biotin nick end labeling
PBS: Phosphate buffered saline
OD: Optical density
ROS: Reactive oxygen species
MDA: Malondialdehyde
SOD: Superoxide dismutase
TBA: Thiobarbituric acid
NBT: Nitroblue tetrazolium
DAB: Diaminobenzidine
IOD: Integrated optical density
oe: Overexpression
GAPDH: Glyceraldehyde-3-phosphate dehydrogenase
WT: Wild type
ANOVA: Analysis of variance
