# Supplementary material for: Omega-3 fatty acids impair miR-1-3p-dependent Notch3 down-regulation and alleviate sepsis-induced intestinal injury
Source: Mol Med. 2022 Jan 28;28:9. doi: 10.1186/s10020-021-00425-w (PMC8796544; doi:10.1186/s10020-021-00425-w)
Supplement: Supplementary file 1 — Additional file 1: Table S1. Primer sequences for RT-qPCR. Note: RT-qPCR, reverse transcription-quantitative polymerase chain reaction; miR-1-3p, microRNA-1-3p; GAPDH, glyceraldehyde-3-phosphate dehydrogenase; F, forward; R, reverse. [file 10020_2021_425_MOESM1_ESM.docx]

**Table S1** Primer sequences for RT-qPCR

| Gene | Sequences |
| --- | --- |
| miR-1-3p | F: 5’-GCGCGTGGAATGTAAAGAAGT-3’ |
|  | R: 5’-AGTGCAGGGTCCGAGGTATT-3’ |
| Notch3 | F: 5’-CCACCTGGTTGCTGCTGATA-3’ |
|  | R: 5’-AGCATAAGTGGGGTGAAGCC-3’ |
| U6 | F: 5’-GTGCTCGCTTCGGCAGCAC -3’ |
|  | R: 5’-AAAAATATGGAACGCTTCA-3’ |
| GAPDH | F: 5’-ATGGTGAAGGTCGGTGTGAAC-3’ |
|  | R: 5’-GGGGTCGTTGATGGCAACA-3’ |

Note: RT-qPCR, reverse transcription-quantitative polymerase chain reaction; miR-1-3p, microRNA-1-3p; GAPDH, glyceraldehyde-3-phosphate dehydrogenase; F, forward; R, reverse.
